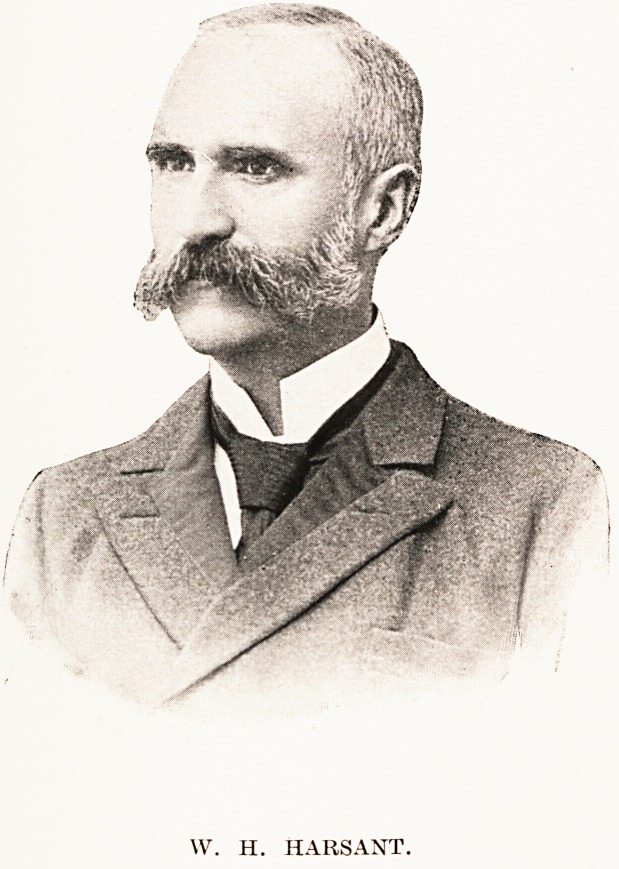# William Henry Harsant

**Published:** 1933

**Authors:** 


### Obituary

### WILLIAM HENRY HARSANT, F.R.C.S.

W. H. HARSANT, who died on 10th February, at his Clifton residence, in his eighty-third year, was born at Epsom, and educated at the City of London School. Entering Guy's Hospital, he qualified as L.S.A. in 1873, the year following he took the M.R.C.S., and was appointed House Surgeon and afterwards Resident Obstetric Officer at Guy's. Leaving London, he was appointed House Surgeon at the Bristol General Hospital, and in 1877 acquired the F.R.C.S. (England).

In 1879 Harsant was appointed Assistant Surgeon on the honorary staff of the Bristol Royal Infirmary, and in the same year initiated a new special department for Diseases of the Ear. He became full Surgeon to the Infirmary in 1885, and resigning in 1902, was appointed Honorary Consulting Surgeon to this institution, which owed him a great debt of gratitude for his twenty-three years of assiduous and painstaking work.

Meanwhile, however, Harsant had made a number of interesting contributions to surgical literature, a bibliography being appended.

He was Lecturer on Anatomy at the Bristol Medical School from 1887, when it was associated with the University College,

W. H. HARSANT.

occupying this important office for six years; his lectures and demonstrations were greatly appreciated by his students. A life-long member of the Bristol Medico-Chirurgical Society, he was on the Journal Committee for several years, taking an active share in the editorial work, and in 1899 was elected to the Presidency of the Society. Although not a prolific writer, Harsant published a number of original contributions on general surgery: he was always most helpful and encouraging to junior colleagues, and in 1894 collaborated with the writer in grafting sheeps' pancreas in a diabetic case with a view to supplying a source of "islet secretion."

An accidental infection of his right hand caused him to lose the index finger. Thenceforth he abandoned his surgical practice, and restricted himself to general practice in Clifton, where he was for many years one of the most respected family advisers. With a peculiarly attractive charm of manner, never failing in quiet courtesy to everyone, the poorest patients and his equals alike, and an innate kindliness and sympathy, he was a man of many friends, beloved by all his patients and warmly esteemed by his fellow-practitioners, and of the many generations of his students.

He married Miss Evans, of Clifton, who predeceased him; and had two daughters, only one of whom survives him.

### BIBLIOGRAPHY OF W. H. HARSANT.

1. "Misplaced Testicles," *Bristol Med.-Chir. Jour.*, 1883, i. 96.

2. "Chancre on the Lip," *Bristol Med.-Chir. Jour.*, 1883, i. 97.

3. "Families with Congenital Syphilis," *Bristol Med.-Chir. Jour.*, 1883, i. 83–87.

4. "Suppuration in the Antrum Cured by Free Drainage," *Bristol Med.-Chir. Jour.*, 1883, i. 93–95.

5. "Lupus Vulgaris," *Bristol Med.-Chir. Jour.*, 1883, i. 88–91.

6. "A Case of Suppurating Middle Ear; Thrombosis and Phlebitis of Lateral Sinus; Death," *Bristol Med.-Chir. Jour.*, 1887, v. 37–42.

7. "Chloroform versus Ether," *Bristol Med.-Chir. Jour.*, 1891, x. 145–154.

8. "Diabetes Treated with Extract and by Grafts of Sheeps' Pancreas," with P. Watson-Williams, *Brit. Med. Jour.*, 1894, ii. 1,303.

9. "Three Cases of Actinomycosis," with G. Smith and J. P. Bush, *The Lancet*, 1897, i. 311.

10. "Cases of Acute Intestinal Obstruction Treated by Operation," *Bristol Med.-Chir. Jour.*, 1900, xviii. 118–122.

## Figures and Tables

**Figure f1:**